# Chromobox homolog 4 overexpression inhibits TNF-α-induced matrix catabolism and senescence by suppressing activation of NF-κB signaling pathway in nucleus pulposus cells: CBX4 overexpression inhibits TNF-α-induced matrix catabolism and senescence

**DOI:** 10.3724/abbs.2022063

**Published:** 2022-06-02

**Authors:** Yangyang Zhang, Shuangxing Li, Junmin Hong, Jiansen Yan, Zhengqi Huang, Jiajun Wu, Zhihuai Deng, Tianyu Qin, Kang Xu, Wei Ye

**Affiliations:** 1 Department of Spine Surgery Sun Yat-sen Memorial Hospital of Sun Yat-sen University Guangzhou 510289 China; 2 Guangdong Provincial Key Laboratory of Malignant Tumor Epigenetics and Gene Regulation Medical Research Center Sun Yat-sen Memorial Hospital Sun Yat-sen University Guangzhou 510289 China; 3 Department of Orthopedics the Seventh Affiliated Hospital of Sun Yat-sen University Shenzhen 518107 China; 4 Department of Orthopedics the Eighth Affiliated Hospital of Sun Yat-sen University Shenzhen 518033 China

**Keywords:** intervertebral disc degeneration, chromobox homolog 4, cell senescence, TNF-α, NF-κB

## Abstract

Intervertebral disc degeneration (IDD) is featured as enhanced catabolism of extracellular matrix (ECM) in the nucleus pulposus (NP), in which tumor necrosis factor-alpha (TNF-α)-related cell senescence is involved. Chromobox homolog protein 4 (CBX4) exhibits anti-inflammatory effects and shows promising therapeutic potential. Thus, in the present study, we explore the role of CBX4 in IDD. Immunohistochemistry staining reveals that CBX4 expression is decreased in severe degenerative NP tissues compared to mild degenerative tissues, and real-time PCR and western blot analysis results show that CBX4 expression is downregulated under TNF-α stimulation in NP cells. siRNA and adenoviruses are used to knockdown or overexpress CBX4, respectively. The results demonstrate that CBX4 knockdown augments the catabolism of ECM in human NP cells, while CBX4 overexpression in rat NP cells restores the ECM degradation induced by TNF-α, as illustrated by immunofluorescence and western blot analysis. In addition, transcriptome sequencing results reveal the regulatory effect of CBX4 on the cell cycle, and further western blot analysis and senescence-associated β-galactosidase staining assay indicate that CBX4 overexpression alleviates cell senescence in the presence of TNF-α. Moreover, the phosphorylation of p65, which indicates the activation of NF-κB signaling, is measured by western blot analysis and immunofluorescence assay, and the results reveal that CBX4 overexpression reduces the TNF-α-induced increase in the p-p65/p65 ratio. In addition, the effect of CBX4 overexpression in NP cells is suppressed by NF-κB agonist. In summary, our results indicate that CBX4 overexpression can suppress TNF-α-induced matrix catabolism and cell senescence in the NP by inhibiting NF-κB activation. This study may provide new approaches for preventing and treating IDD.

## Introduction

Low back pain (LBP) is considered as one of the leading causes of disability worldwide, especially in elderly individuals, and is related to high costs [ 1– 3] . Factors that contribute to LBP include aging [4], mechanical stress [5], injury [6], and gene susceptibility [7], while age-related degeneration of intervertebral discs (IVDs) and facets lead to most cases of nonmechanical LBP [8]. Attention should be paid to LBP because over one in five individuals in the world will be over 60 years old, as suggested by demographic projections, indicating the great importance of LBP prevention [9].

Intervertebral disc degeneration (IDD) occurs as IVD components alter with aging [10]. The loss of aggrecan (ACAN) and type II collagen (COL2A1) in the nucleus pulposus (NP) is one of the characteristics of IDD, which is mainly caused by disturbances in matrix metabolism, such as reduction in matrix synthesis and increase in matrix degradation [11]. Upregulation of matrix metalloproteinases (MMPs) and a disintegrin and metalloproteinase with thrombospondin motif proteins (ADAMTSs) caused by proinflammatory factors such as tumor necrosis factor-alpha (TNF-α) damages the extracellular matrix (ECM), compromises the integrity of the NP, and ultimately leads to biomechanical dysfunction of the IVD [ 12, 13] . Therefore, finding an approach to alleviate ECM degradation in the NP is of great importance.

Chromobox homolog (CBX) proteins play vital roles in epigenetically regulating embryonic development in mammals [14] because these proteins help maintain the self-renewal of stem cells [15] and contribute to cancers such as colorectal cancer [16] and hepatocellular carcinoma [17]. CBX8 participates in the ECM metabolism through regulating cell proliferation and cell cycle in NP cells, another CBX protein member, CBX4, could alleviate osteoarthritis by restraining human mesenchymal stem cell senescence [ 18, 19] . In addition, UNC3866, a new chemical probe targeting CBX4 and CBX4-7, could regulate cell proliferation [20]. Therefore, the effect of CBX4 in IDD is worth exploring.

Cell senescence, which is defined as the arrest state in the cell cycle, is a hallmark of aging [ 21– 23] . Previous studies have demonstrated accelerated cell senescence in degenerative IVD cells, and shown that inhibiting cell senescence in NP could be one approach for alleviating IDD [ 24, 25] . Moreover, CBX4 was reported to regulate senescence in different types of cells, such as human epidermal stem cells and human mesenchymal stem cells [ 19, 26] , which motivated further investigation into whether CBX4 participates in IDD by modeling cell senescence in NP cells.

Additionally, the nuclear factor-kappa B (NF-κB) signaling pathway, one of the classic pathways participating in the process of inflammation, plays important roles in regulating cell senescence in different types of tissues [ 27– 29] . Previous studies revealed that chemicals such as resveratrol could protect NP cells from TNF-α-induced senescence by inhibiting this pathway [30], whereas the role of CBX4 in the NF-κB pathway remains unclear. Thus, whether NF-κB signaling is involved in CBX4-mediated ECM regulation in NP needs further clarification.

In the present study, we hypothesize that CBX4 regulates the process of IDD by inhibiting TNF-α-induced cell senescence and ECM catabolism through suppressing NF-κB activation.

## Materials and Methods

### Cell isolation and culture

The experiments were approved by the Ethics Committee of Sun Yat-sen Memorial Hospital, Sun Yat-sen University (No. [2020]-405), and the Animal Care and Use Committee of Sun Yat-sen University (No. SYSU-IACUC-2020-B0933). Rat NP tissue was collected, cut into pieces and digested into cells which were then cultured in DMEM (HyClone, Logan, USA) with 10% FBS (Gibco, New York, USA), penicillin G in a humidified cell incubator (Thermo Fisher Scientific, Waltham, USA). Human NP cells were obtained from ScienCell (ScienCell, San Diego, USA) with authentication. NF-κB activator 1 (MedChemExpress, Princeton, USA) was added after TNF-α (Novoprotein, Suzhou, China) stimulation of rat NP cells.

### Human tissue collection and IHC

Human NP tissue was obtained from patients who underwent lumbar interbody fusion for degenerative disc diseases, and the procedures were approved by the Medical Ethics Committee of Sun Yat-sen Memorial Hospital, Sun Yat-sen University. From September 2020 to March 2021, 10 disc samples (male:female=3:7) were collected, evaluated by Pfirrmann classification and classified into 2 groups.

Human tissues paraffin sections were deparaffinized with xylene and rehydrated, followed by retrieval with EDTA buffer (ZSGB-BIO, Beijing, China) for 10 min at 100°C. Hydrogen peroxide (3%) was used to quench endogenous peroxidase activity. Then, goat serum (ZSGB-BIO) was used to block the paraffin sections for 30 min. The paraffin sections were incubated with PBS or anti-CBX4 (1:100; Abcam, Cambridge, UK) primary antibodies overnight at 4°C. After being incubated with biotin-labeled secondary antibodies for 30 min at room temperature and HRP-conjugated streptavidin for 20 min, the sections were developed with DAB solution (ZSGB-BIO), and counterstained with 1% hematoxylin. Lastly, sections were mounted, photographed under a microscope (Nikon, Tokyo, Japan).

### Western blot analysis

Stimulated NP cells were collected and then lysed with RIPA lysis buffer (CWBIO, Beijing, China) for total protein, or with an extraction kit for cytoplasmic and nuclear protein (CWBIO). After being centrifuged and collected, the supernatant containing proteins was then quantified using a BCA assay kit (CWBIO). Afterwards, the proteins were separated by SDS-PAGE and electro-transferred to polyvinylidene difluoride membranes (Millipore, Darmstadt, Germany). After being blocked with 5% bovine serum albumin in TBST for 1 h, the membranes were then incubated at 4°C overnight with anti-CBX4 (1:1000; Abcam), anti-MMP3 (1:1000; Abcam), anti-MMP13 (1:1000; Abcam), anti-ADAMTS5 (1:1000; Abcam), anti-COX2 (1:1000; Abcam), anti-COL2A1 (1:1000; Abcam), anti-P53 (1:1000; Abcam), anti-P21 (1:1000; Abcam), anti-β-actin (1:1000; Abclonal, Wuhan, China), anti-p-p65 (1:1000; Abclonal), anti-p65 (1:1000; Abclonal), or anti-Histone H3 (1:1000; CST, Danvers, USA) antibodies. Afterwards, the membranes were incubated with the corresponding HRP-conjugated secondary antibodies (1:8000; Abclonal). The protein bands were visualized using an Enhanced Chemiluminescence kit (Vazyme, Nanjing, China) and then quantified by Image J (National Institutes of Health, Bethesda, USA).

### Cell β-gal staining

NP cells were inoculated in a 6-well culture plate and cultured to 70% confluence. After the corresponding treatment, cells were fixed with β-gal fix solution (Beyotime Biotechnology, Shanghai, China). Then cells were incubated with senescence-associated beta-galactosidase (SA-β-gal) staining solution (Beyotime Biotechnology) overnight at 37°C free from CO _2_, followed by analysis by microscopy under a light microscope (Olympus, Tokyo, Japan).

### EdU incorporation assay

EdU incorporation assay was performed using BeyoClick™ EdU-488 test kit (Beyotime Biotechnology). Briefly, NP cells were inoculated in a 6-well culture plate and cultured to 70% confluence. After the corresponding treatment, cells were incubated with EdU working fluid for 2 h. Then, the culture medium was removed and 1 mL fixing solution was added to each well and incubated at room temperature for 15 min. After removal of the fixing solution, fixed cells in each well were washed with 1 mL of washing solution for 3 times, followed by treatment with permeabilization solution. After 3 times wash, 0.5 mL of Click reaction solution was added to each well and incubated for 30 min. Then, cells were washed for 3 times and the cell nuclei were stained with Hoechst. Finally, cells were examined under a fluorescence microscope (Olympus).

### Immunofluorescence microscopy

NP cells were inoculated in a 24-well culture plate and cultured to 70% confluence. Cells were fixed with 4% paraformaldehyde fix solution and permeabilized with 0.5% Triton X-100. Goat serum was used to block the non-specific binding sites before the specimen were incubated with an anti-p65 (1:200; Abclonal), anti-CBX4 (1:200; Abcam), or anti-COL2A1 (1:200; Abcam) antibodies overnight at 4°C, followed by incubation with an Alexa Fluor 488-conjugated anti-rabbit IgG secondary antibody (ZSGB-BIO) for 1 h at room temperature. The cell nuclei were stained with DAPI (Solarbio) or Hoechst (Solarbio). Afterwards, the immunofluorescence signals of proteins were detected with a fluorescence microscope (Olympus).

### CBX4 knockdown with siCBX4 in human NP cells

NP cells were transfected with siRNA against CBX4 using Lipofectamine 2000 (Invitrogen, Carlsbad, USA) transfection reagent. siCBX4 was synthesized by RiboBio (Guangzhou, China) with different target sequences: siCBX4-1, 5′-GACGCATCGTGATCGTGAT-3′, siCBX4-2, 5′-GCAAGAGCGGCAAGTACTA-3′, and siCBX4-3, 5′-CCGTTACTTTCAAGGAGTA-3′. A mock control siCTR was also obtained from RiboBio (Cat number: siQ0002).

### CBX4 overexpression with Ad-CBX4 in rat NP cells

To overexpress CBX4, rat NP cells were transfected with an adenovirus carrying a recombinant CBX4 plasmids and free plasmids constructed by Hanbio Biotechnology (Shanghai, China). After transfection for 8 h, the culture medium with plasmids was removed, and western blot analysis was performed to demonstrate the effects of recombinant CBX4 plasmids.

### Transcriptome sequencing

For RNA sequencing, total RNA was extracted using Trizol (TaKaRa, Dalian, China) from 4 groups of rat NP cells, *i*. *e*., control cells, CBX4-overexpressing cells, TNF-α-stimulated cells, and CBX4-overexpressing cells stimulated with TNF-α. Then cDNAs were generated using the RNA-Seq sample preparation kit (Illumina, San Diego, USA) and analyzed on Illumina Illumina Hiseq2500 platform (Lianchuan Biotech, Hangzhou, China). The sequencing data were subject to GO and KEGG database. According to the distribution, results of the significant enrichment analysis were sorted by the GO term and KEGG pathway. The significantly enriched pathways were determined when *P*<0.05.

### Statistical analysis

The results were obtained from at least three independent experiments. If normally distributed, the data are analyzed by Student’s *t* test or ANOVA, otherwise, by Mann-Whitney test or Kruskal-Wallis test. Data were presented as the mean±SD. *P*<0.05 was considered statistically significant.

## Results

### CBX4 expression was reduced in degenerated human NP tissues

**Figure 1:**
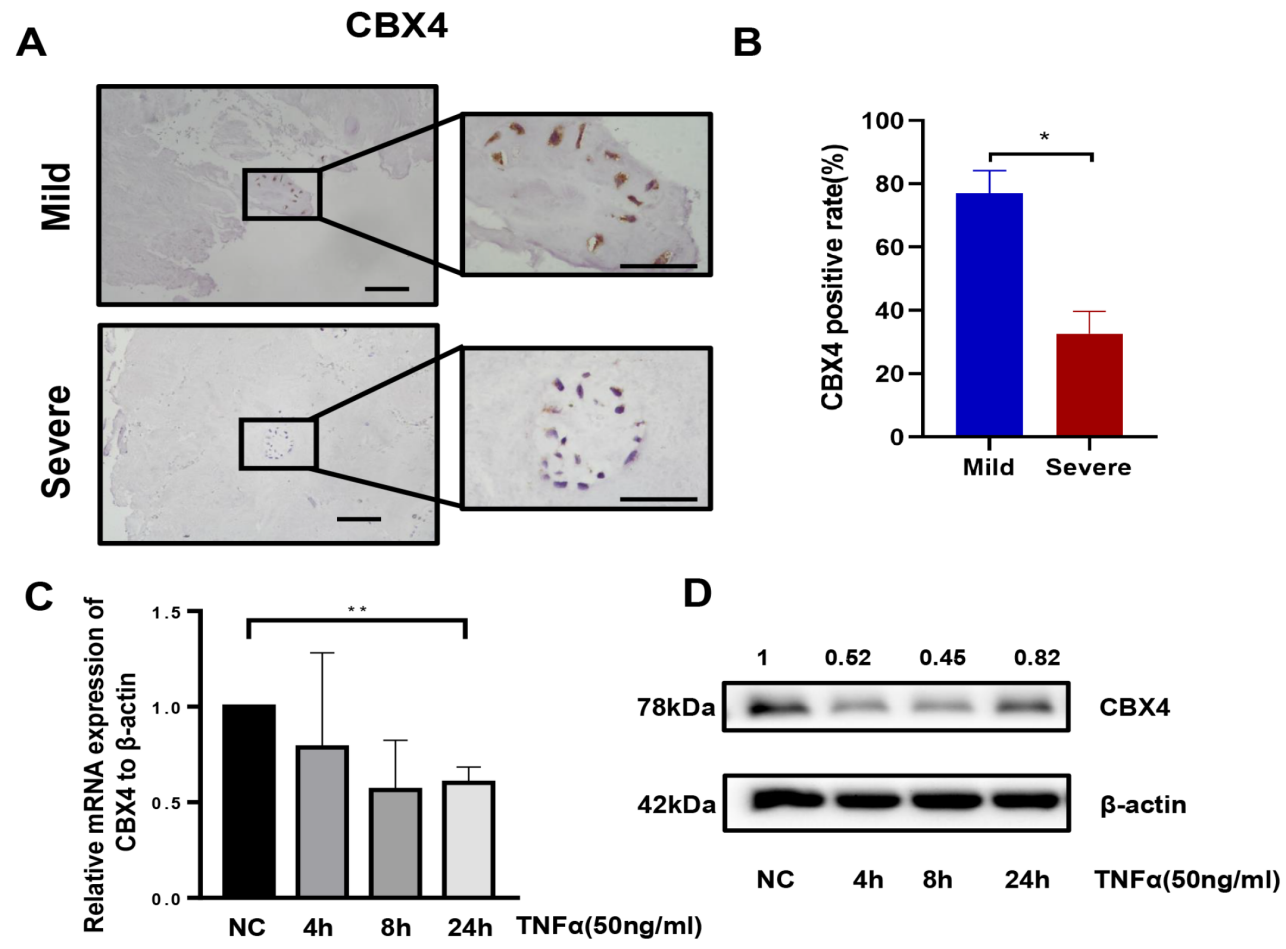
Figure 1 CBX4 expression was reduced in degenerated human NP tissues (A,B) Representative immunohistochemical images showing CBX4 in human IVDs from mild and severe cases. Western blot analysis revealed that CBX4 protein expression could be inhibited by TNF-α in a dose-dependent manner (C) and time-dependent manner (D). Data were presented as the mean±SD of three independent experiments. * P<0.05, ** P<0.01.

To investigate CBX4 expression in mildly and severely degenerated human NPs, we collected human NP tissues of different degrees for immunohistochemistry staining and divided them into mild (Pfirrmann grade III) and severe (Pfirrmann grade IV–V) groups. CBX4 expression was markedly reduced in the severe group compared to that in the mild group ( Figure 1A,B). To verify the effect of TNF-α on CBX4 expression in NP cells, stimulation with TNF-α (50 ng/ml) for 4, 8 and 24 h was conducted, and the results demonstrated that TNF-α markedly reduced the expression of CBX4 ( Figure 1C,D). TNF-α exacerbated IDD, while CBX4 might alleviate TNF-α-related IDD.

### CBX4 mitigated matrix degradation in NP cells

**Figure 2:**
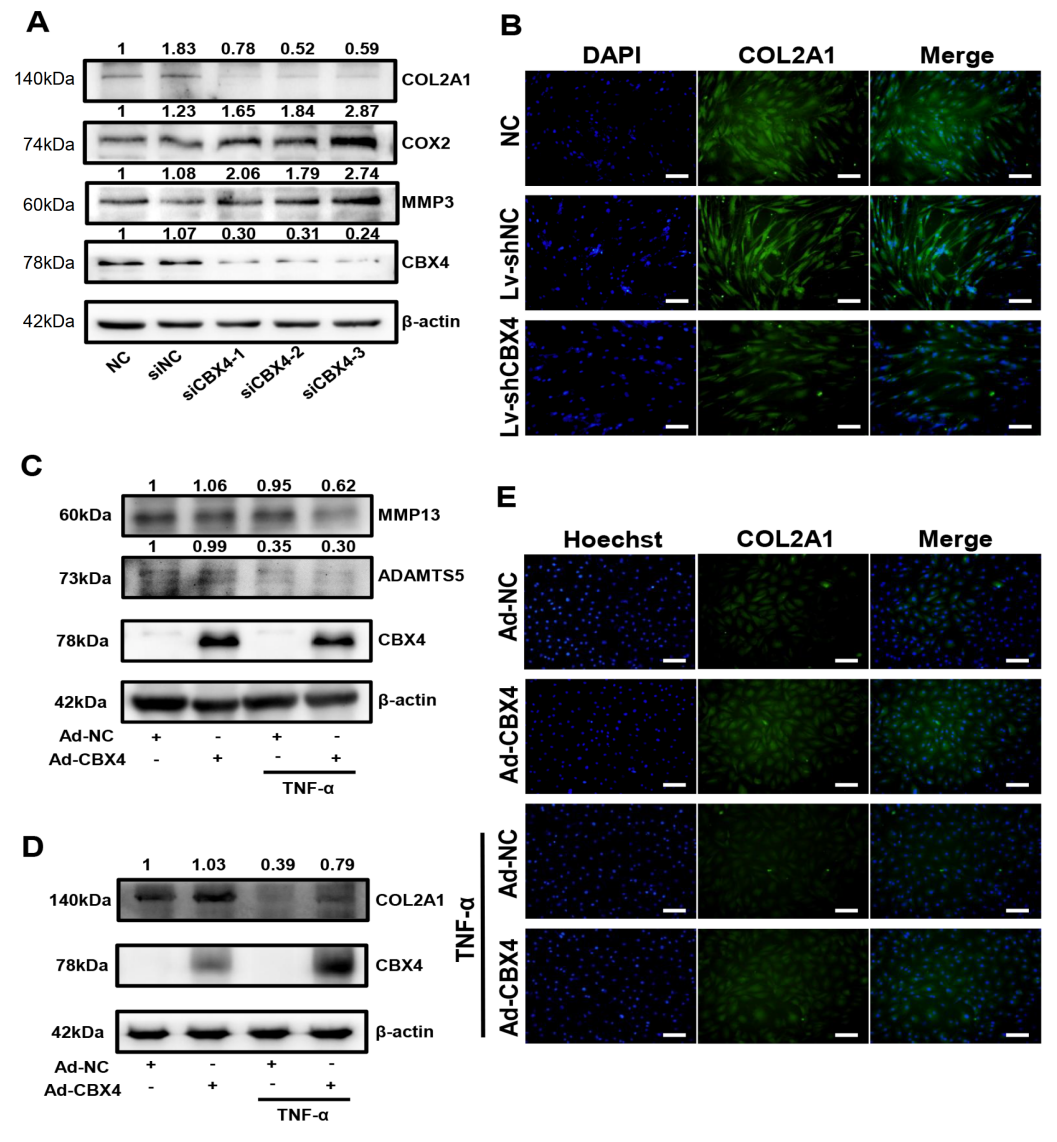
Figure 2 CBX4 regulated matrix metabolism in NP cells (A) Western blot analysis revealed that CBX4 knockdown inhibited the expression of CBX4 and COL2A1 but enhanced the expressions of MMP3 and COX2. (B) Immunofluorescence staining revealed that CBX4 knockdown inhibited the expression of COL2A1. (C,D) Western blot analysis showed that CBX4 overexpression reversed the impact of TNF-α: CBX4 expression was significantly enhanced after transfection, MMP3 and ADAMTS5 expressions were increased and COL2A1 expression was reduced. (E) Immunofluorescence staining revealed that CBX4 overexpression promoted the expression of COL2A1 in the presence of TNF-α. Three independent experiments were performed. Scale bar=100 μm.

To reveal the role of CBX4 in matrix metabolism, western blot analysis and immunofluorescence assay were conducted in NP cells. When CBX4 was knocked down by siCBX4 in human NP cells, the expressions of MMP3 and COX2 were enhanced, while the expression of COL2A1 was significantly suppressed ( Figure 2A,B). Consistently, in the presence or absence of TNF-α, CBX4 overexpression reduced the expressions of MMP3 and ADAMTS5 in rat NP cells ( Figure 2C), while the expression of COL2A1 was markedly enhanced, as shown by western blot analysis and immunofluorescence ( Figure 2D,E). These results revealed that CBX4 mitigated matrix degradation in NP cells.

### The potential role of CBX4 in rat NP cells

**Figure 3:**
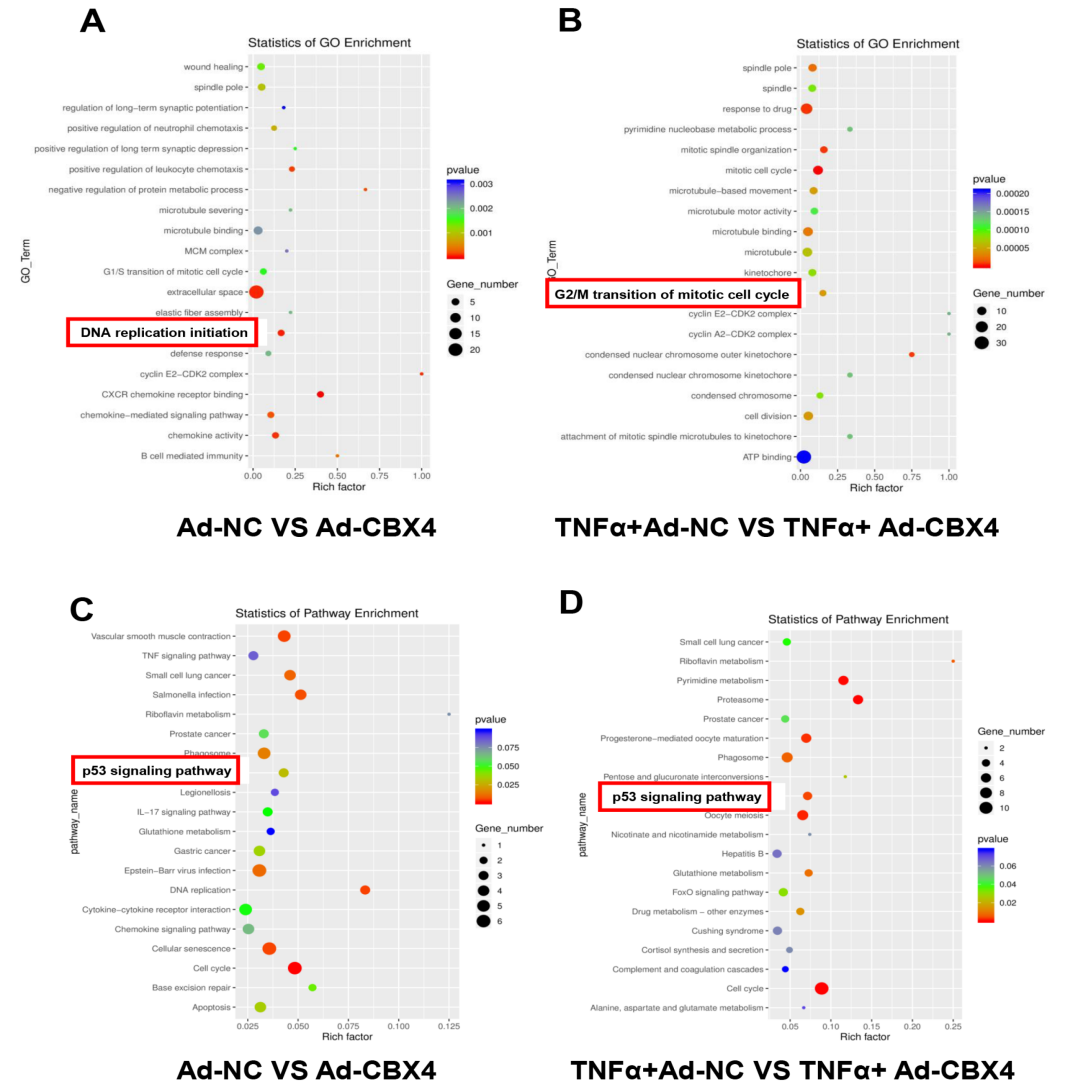
Figure 3 CBX4 regulated the senescence and proliferation of NP cells (A,B) GO analysis of the differentially expressed genes (CBX4 overexpression versus control and CBX4 overexpression+TNF-α versus TNF-α). (C,D) KEGG pathway analysis of the differentially expressed genes (CBX4 overexpression versus control and CBX4 overexpression+TNF-α versus TNF-α).

To explore the effect and potential mechanism of CBX4, transcriptome sequencing was then conducted in NP cells with or without TNF-α and CBX4 overexpression. Differentially expressed genes (CBX4 overexpression versus control, and CBX4 overexpression with TNF-α stimulation versus TNF-α stimulation only) were identified. GO functional annotation analysis showed that G1/S transition of mitotic cell cycle, DNA replication initiation, and G2/M transition of mitotic cell cycle were significantly related to CBX4 in NP cells ( Figure 3A,B), suggesting that CBX4 participated in the proliferation of NP cells. In addition, KEGG pathway enrichment analysis revealed that these differentially expressed genes mainly participated in the p53 signaling pathway, DNA replication, cellular senescence, and the cell cycle ( Figure 3C,D).

### CBX4 inhibited senescence and promoted proliferation in rat NP cells

**Figure 4:**
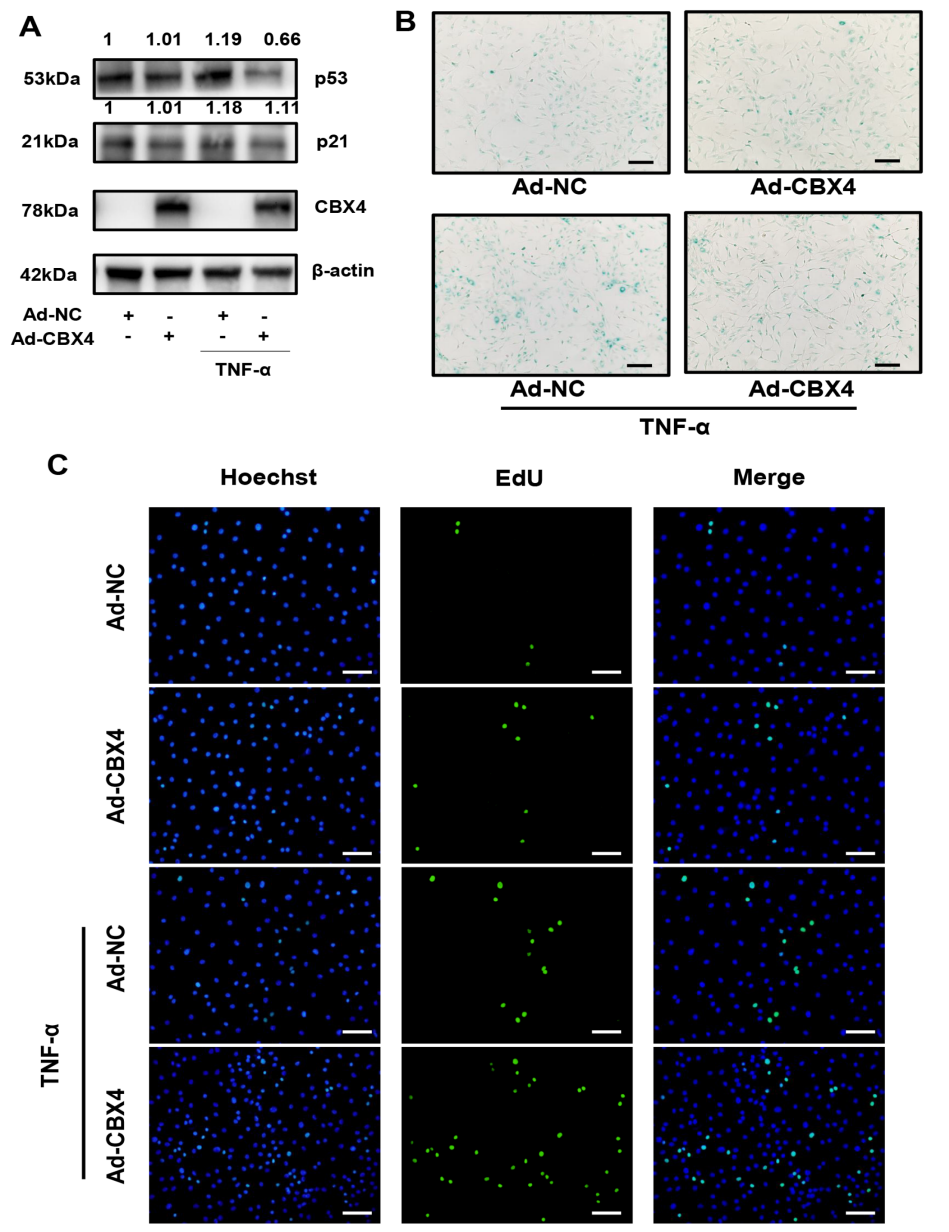
Figure 4 CBX4 participated in senescence and proliferation in rat NP cells (A) Western blot analysis revealed that CBX4 overexpression increased the expression of CBX4 and inhibited the expressions of p53 and p21 with or without TNF-α. (B) β-Gal staining revealed that CBX4 overexpression suppressed TNF-α-induced senescence. (C) EdU staining showed that CBX4 overexpression promoted the proliferation of NP cells. Three independent experiments were performed. Scale bar=100 μm.

Consistent with the results of the bioinformatics analysis, the experimental results revealed that CBX4 participated in the senescence and proliferation of rat NP cells. As shown in Figure 4A, the protein expression levels of p53 and p21 were increased by TNF-α, but reversed significantly by CBX4 overexpression. In addition, SA-β-gal-positive cells were reduced by CBX4 overexpression with or without TNF-α, which confirmed that CBX4 overexpression alleviated TNF-α-induced senescence ( Figure 4B). Moreover, there were more EdU-positive cells in the CBX4 overexpression group than those in the control group, revealing that CBX4 overexpression promoted the proliferation of NP cells ( Figure 4C).

### CBX4 regulated matrix metabolism and senescence in rat NP cells via suppressing NF-κB activation

**Figure 5:**
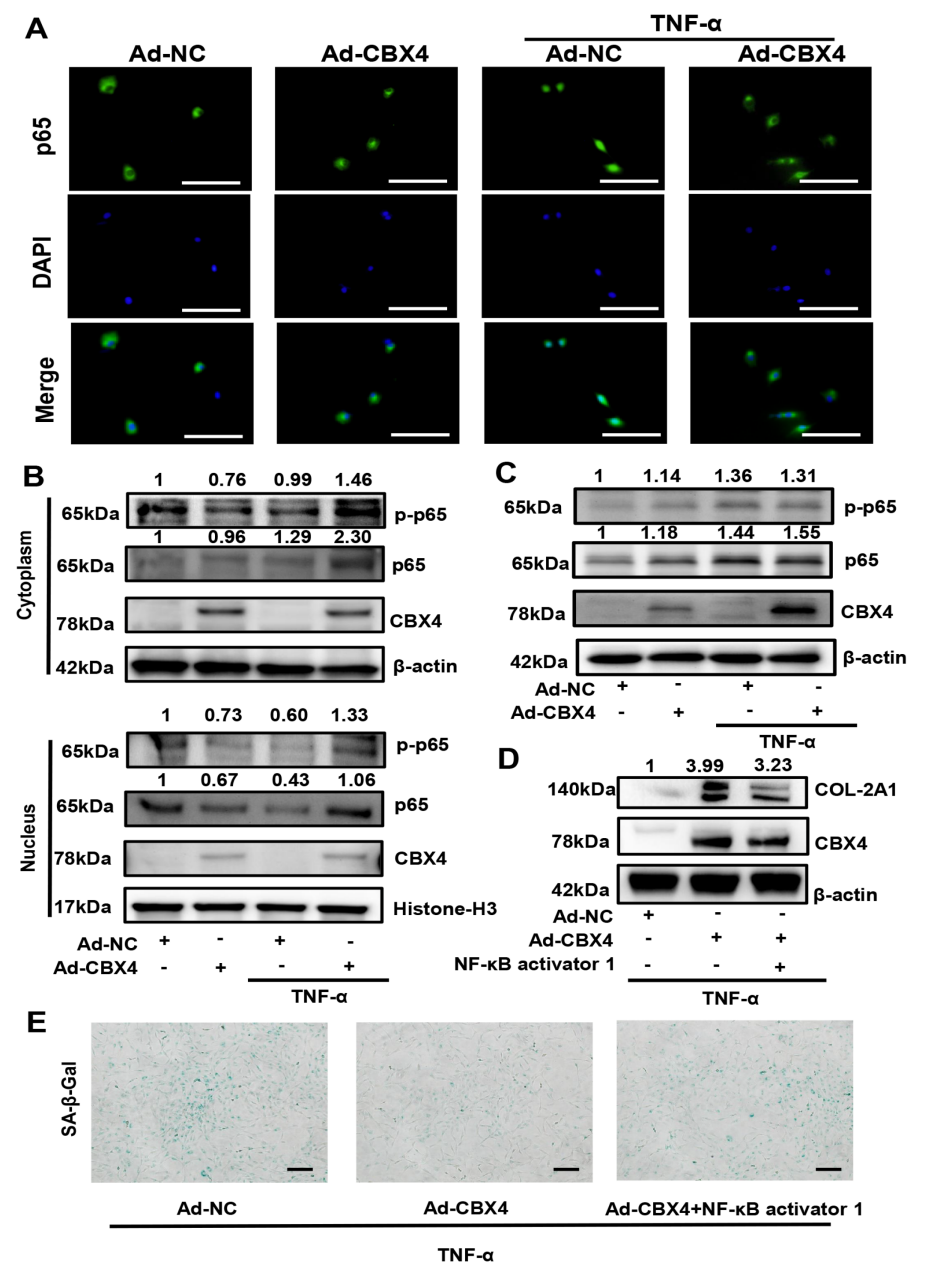
Figure 5 CBX4 overexpression regulated matrix metabolism and senescence by suppressing the activation of the NF-κB pathway in rat NP cells (A) Immunofluorescence staining demonstrated that CBX4 overexpression suppressed the nuclear transfer of phosphorylated P65 after TNF-α stimulation. (B) Western blot analysis revealed that CBX4 overexpression increased the expression of CBX4 and inhibited the phosphorylation of p65 in the nucleus. (C) Western blot analysis demonstrated that CBX4 overexpression increased the expression of CBX4 and suppressed the p-p65/p65 ratio after TNF-α stimulation. (D) Western blot analysis showed that CBX4 expression was significantly enhanced after tranfection and NF-κB agonist could suppress the effect of CBX4 overexpression on matrix metabolism. (E) SA-β-Gal staining demonstrated that CBX4 suppressed senescence in the presence of TNF-α, as limited by NF-κB agonist. Three independent experiments were performed. Scale bar=100 μm.

It was reported that TNF-α enhanced NF-κB activation in IDD [29]. Therefore, to reveal the relationship between CBX4 and NF-κB signal pathway, further experiments were performed on the NF-κB signaling. Our immunofluorescence assay results showed that CBX4 significantly inhibited the nuclear translocation of NF-κB in the presence of TNF-α ( Figure 5A). Moreover, the nuclear protein expression of phosphorylated p65 was increased by TNF-α, which however was significantly reversed by CBX4 overexpression ( Figure 5B). In addition, without TNF-α stimulation, CBX4 had no impact on p65 phosphorylation, while p65 phosphorylation was significantly inhibited by CBX4 overexpression in the presence of TNF-α ( Figure 5C). Furthermore, as shown in Figure 5D, CBX4 overexpression elevated the expression of COL-2A1, which was suppressed by NF-κB agonist. SA-β-Gal staining results confirmed that NF-κB agonist also inhibited the effect of CBX4 overexpression on cell senescence ( Figure 5E).

## Discussion

In the present study, we demonstrated that CBX4 expression was downregulated in severely degenerative human NP tissues compared with mildly degenerative ones. Knockdown of CBX4 in NP cells promoted ECM degradation, while CBX4 overexpression alleviated the degradation of ECM in the presence of TNF-α. Transcriptome sequencing and further experiments showed that CBX4 overexpression could suppress TNF-α-induced cell senescence. Moreover, NF-κB activation played an important role in CBX4 suppressing NP cell senescence under TNF-α stimulation.

IDD is characterized by a disturbance in matrix metabolism in NP cells, and MMPs, such as MMP3, important enzymes that exacerbate ECM degradation [31]. CBX proteins play important roles in maintaining cellular homeostasis during embryonic development in mammals, and contribute to different types of cancers [ 14– 17] . CBX8 participates in IDD, and CBX4 exhibits therapeutic potential in treating degenerative diseases such as osteoarthritis [19]. In the present study, CBX4 expression was examined in human NP tissue and the results revealed a reduction in CBX4 in severe degenerative NP tissues compared with that in mild degenerative ones. Additionally, knockdown of CBX4 in human NP cells promoted the expressions of COX2 and the matrix-degrading enzyme MMP3, and suppressed the expression of COL2A1, while overexpression of CBX4 restored the expression of COL2A1 in the presence of TNF-α. These results indicated that CBX4 participated in IDD process, and that CBX4 overexpression could be a protective approach to prevent IDD.

It was reported that CBX4 regulates cellular homeostasis in multiple ways, such as by regulating the DNA repair process and modifying histones, and it functions as one of the SUMOylation-related enzymes [ 18, 32, 33] . Therefore, in the present study, transcriptome sequencing was carried out to determine how CBX4 functions in the process of IDD, and our results showed that CBX4 overexpression with or without TNF-α caused different expression levels of genes involved in the cell cycle regulation.

Cell senescence is defined as a stable arrest state in the cell cycle [21]. As previously reported, inflammatory environments could accelerate senescence in NP cells, and senescent cells tended to generate more proinflammatory cytokines, such as TNF-α and interleukin-1 beta (IL-1β), indicating an close relation between cell senescence and inflammatory environments [ 34, 35] . In addition, cell senescence was discovered in different types of tissues, including bone marrow, brain and blood [23], and modulating cell senescence could prevent IDD [ 25, 26] . In the current study, we demonstrated that TNF-α promoted the NP cell senescence, as shown by western blot analysis and SA-β-Gal experiments, while CBX4 overexpression alleviated this effect. These results indicated that CBX4 might alleviate IDD by suppressing TNF-α-induced cell senescence.

Previous studies have shown an upregulation in the p-p65/p65 ratio as IDD proceeds, and the phosphorylation of p65 is considered the hallmark of NF-κB signaling pathway activation [36]. Moreover, it was reported that multiple chemicals regulate cell senescence in the NP through the NF-κB pathway [ 30, 37] , but whether CBX4 is involved in NF-κB regulation remains to be uncovered. In the current study, p65 phosphorylation in both the cytoplasm and nucleus in NP cells was examined, and the results demonstrated that CBX4 overexpression could reduce the p-p65/p65 ratio in the nucleus in NP cells. In addition, NF-κB agonist could suppress the effect of CBX4 overexpression on matrix metabolism and senescence in NP cells. Thus, we conclude that NF-κB activation participates in CBX4-mediated regulation of the IDD process.

**Figure 6:**
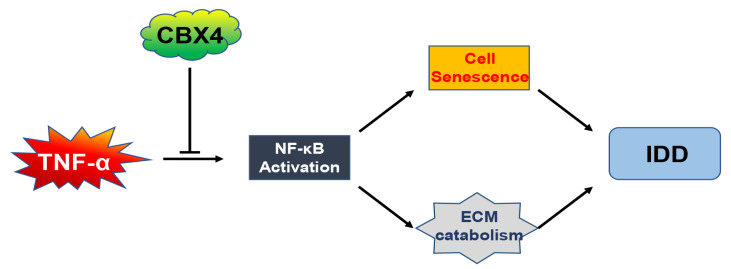
Figure 6 Schematic diagram of the role of CBX4 in NP cells CBX4 inhibits TNF-α-induced matrix catabolism and senescence by suppressing the activation of the NF-κB signaling pathway in nucleus pulposus cells.

In summary, in the present study we reveal that CBX4, one of the CBX proteins, regulates ECM homeostasis and senescence in NP cells, in which NF-κB/p65 plays an important role ( Figure 6). Targeting CBX4 may provide new strategies for the prevention as well as the treatment of IDD. Further *in vivo* verification using animal models is worth investigating.
